# Pancreaticoduodenal artery aneurysm presenting with retroperitoneal haemorrhage and gastric outlet obstruction: a case report

**DOI:** 10.1093/jscr/rjag263

**Published:** 2026-04-15

**Authors:** Su Su (Shannon) Naing

**Affiliations:** The Redland Bay Hospital, General Surgical Department, 21 Weippin St, Cleveland, QLD 4163, Australia

**Keywords:** Pancreaticoduodenal artery, retroperitoneal haematoma

## Abstract

Pancreaticoduodenal artery (PDA) aneurysms are rare visceral vascular lesions, accounting for less than 2% of all visceral artery aneurysms. They are frequently associated with celiac artery stenosis, which promotes high-flow collateralization through the pancreaticoduodenal arcade. We present the case of a 73-year-old man who presented with acute abdominal pain and syncope. Initial imaging revealed a large retroperitoneal hematoma without active contrast extravasation. The patient subsequently developed gastric outlet obstruction due to extrinsic compression by the hematoma. Repeat CT angiography identified a PDA aneurysm associated with severe celiac trunk stenosis. The patient was successfully treated with transcatheter coil embolization. This case highlights the diagnostic challenges of PDA aneurysms and the importance of evaluating the celiac axis in patients with retroperitoneal hemorrhage.

## Introduction

Visceral artery aneurysms are uncommon clinical entities, with pancreaticoduodenal artery (PDA) aneurysms comprising only 2% of these cases [1]. Despite their rarity, they carry a disproportionately high risk of rupture, which is independent of aneurysm size. A strong association exists between PDA aneurysms and celiac artery stenosis, where hemodynamic alterations and increased collateral flow contribute to aneurysm formation [2]. Rupture can lead to life-threatening retroperitoneal or intraperitoneal hemorrhage, with mortality rates reported as high as 50% [3]. This report discusses a case of a ruptured PDA aneurysm complicated by gastric outlet obstruction, emphasizing the need for a high index of suspicion and appropriate vascular imaging.

## Case presentation

**Figure 1:**
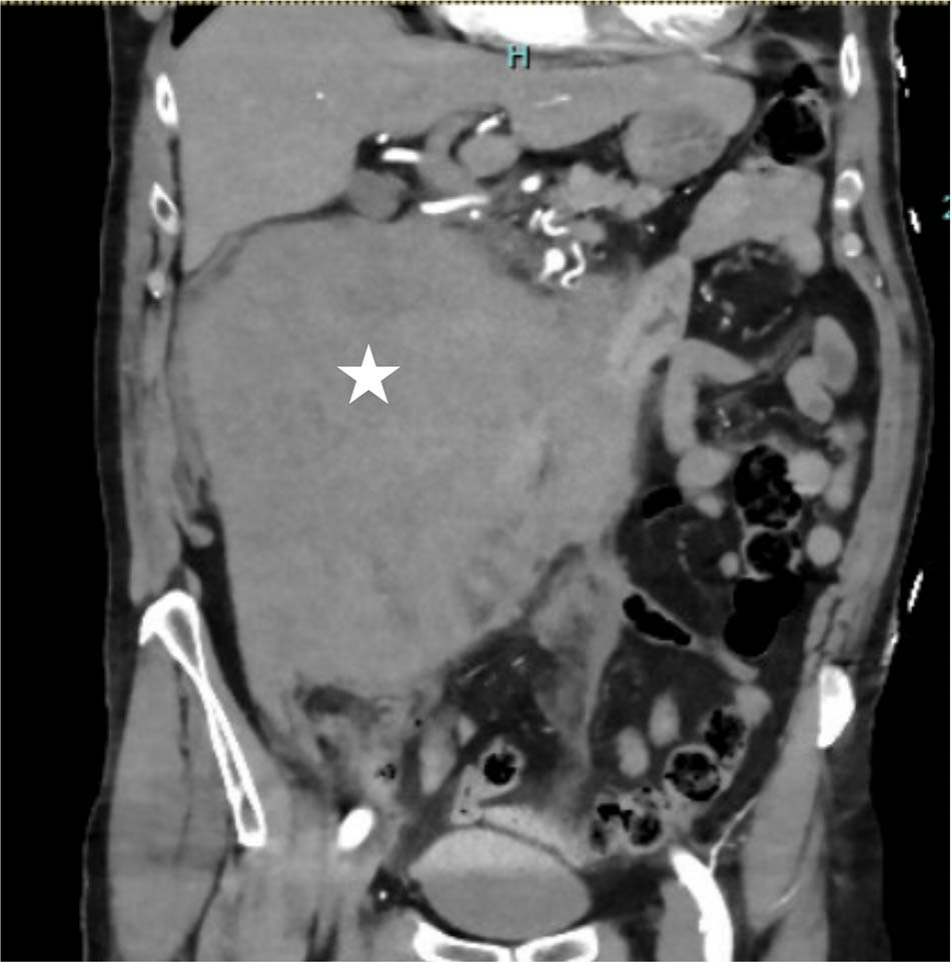
Coronal contrast-enhanced CT demonstrating a massive right-sided retroperitoneal hematoma (asterisk).

A 73-year-old man presented to the Emergency Department with acute onset right-sided abdominal pain and a syncopal episode. He has no significant past medical history and is not on anticoagulation. On presentation, he was hemodynamically stable with heart rate of 70, blood pressure 113/75, respiratory rate 12, oxygen saturation 95% on room air and afebrile. His hemoglobin (Hb) is 119 and coagulation studies were normal. The computed tomographic (CT) angiography (Fig. 1) of the abdomen demonstrated a large retroperitoneal hematoma measuring 19 cm in width. No active extravasation was identified on the arterial phase. He was managed conservatively with observation and was discharged after three days as he was clinically stable with no change in Hb level.

**Figure 2:**
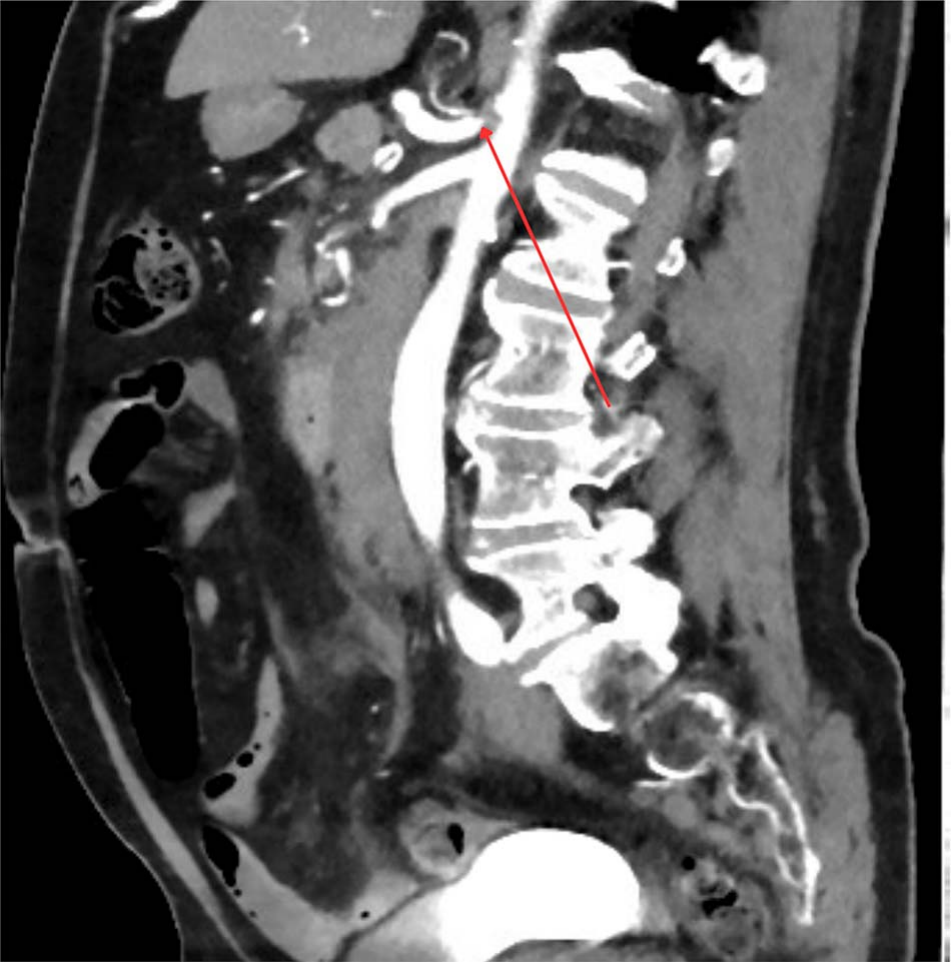
Sagittal contrast-enhanced CT image demonstrating significant stenosis at the origin of the celiac axis. The site of the narrowing is marked by arrow.

Three days post-discharge, the patient re-presented to the hospital with persistent vomiting and oral intolerance, consistent with gastric outlet obstruction. His repeat Hb is also stable with 119 and normal INR. The repeat CT was performed, which revealed extrinsic compression of the duodenum by the organizing hematoma and duodenitis noted. He was transferred to a tertiary center for further management. Nasogastric tube was inserted for gastric decompression. The endoscopy was performed the next day which showed normal endoscopic finding and naso-jejunostomy tube was placed for feeding. Within 3 days of admission, his Hb has slowly down trending to 99 and therefore, CT multiphase scan was arranged. Crucially, the CT scan identified tight stenosis of the celiac trunk (Fig. 2) and a distinct aneurysm within the pancreaticoduodenal artery arcade.

**Figure 3:**
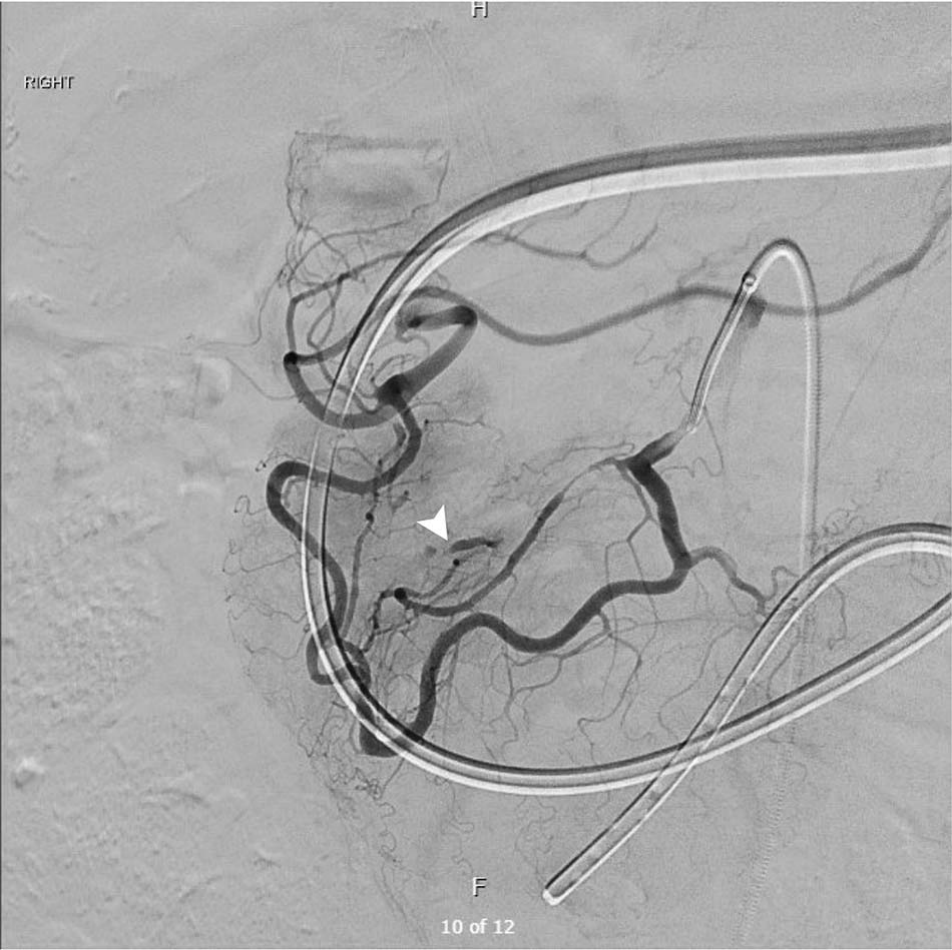
Digital subtraction angiogram demonstrating the hypertrophied pancreaticoduodenal arcade providing collateral retrograde flow from the SMA to the celiac territory. A distinct aneurysm (white arrowhead) is visualized arising from the collateral pathway, confirming the diagnosis.

The patient underwent catheter angiography, confirming the presence of the aneurysm within the collateral pathway (Fig. 3). He underwent successful transcatheter embolization of the pancreaticoduodenal aneurysm. His post-procedural recovery was uneventful, and his obstructive symptoms resolved over a course of 2 weeks as the hematoma burden decreased. Ultrasound Doppler of mesenteric vessels showed more than 75% coeliac axis stenosis proximally. He is currently under surveillance by the vascular outpatient clinic with three yearly CT angiography for recurrence of aneurysms/pseudoaneurysms and would consider for elective celiac axis stenting if recurs.

## Discussion

Aneurysms of the pancreaticoduodenal artery are rare but clinically significant due to their propensity for rupture. Unlike other visceral aneurysms, where size is a predictor of rupture risk, PDA aneurysms are prone to rupture regardless of diameter [4].

The etiology of PDA aneurysms is distinct; they are true aneurysms often secondary to hemodynamic stress. Approximately 60%–80% of cases are associated with celiac artery stenosis or occlusion (e.g. due to median arcuate ligament syndrome or atherosclerosis) [2, 5]. This obstruction necessitates retrograde blood flow from the superior mesenteric artery (SMA) to the celiac territory via the pancreaticoduodenal arcade. The resulting high-flow, high-pressure state causes arterial dilation and aneurysm formation [5].

The clinical presentation varies from incidental radiological findings to life-threatening shock. In this case, the patient presented with a large retroperitoneal hematoma. A notable complication observed here was gastric outlet obstruction (GOO). While rare, large retroperitoneal hematomas can exert sufficient mass effect on the fixed duodenum to cause obstruction, necessitating gastric decompression and nutritional support while the hematoma resorbs [6]. Diagnostic imaging is pivotal. CT angiography is the modality of choice, allowing visualization of the aneurysm, the extent of hematoma, and the patency of the celiac axis.

Therapeutic management has shifted from open surgery to endovascular therapy. Transcatheter coil embolization is now the first-line treatment for both ruptured and unruptured PDA aneurysms, offering high success rates with lower morbidity than pancreatoduodenectomy or ligation [7]. However, embolization addresses the aneurysm but not the underlying high-flow state. Therefore, revascularization of the celiac trunk (via stenting or surgical bypass) is often considered to reduce collateral flow and prevent recurrence, although this remains a subject of debate and is decided on a case-by-case basis [8].

## Conclusion

Pancreaticoduodenal artery aneurysms are rare, high-risk lesions often associated with celiac axis stenosis. Clinicians must maintain a high index of suspicion for this pathology in patients presenting with spontaneous retroperitoneal hemorrhage. Transcatheter embolization is the preferred initial treatment, with long-term follow-up required to monitor for recurrence or the need for celiac revascularization.
